# Trypanocidal activity of methanol extracts of the hemolymph of *Sarcophaga argyrostoma* larva against *Trypanosoma evansi* infected mice

**DOI:** 10.14202/vetworld.2020.1599-1604

**Published:** 2020-08-15

**Authors:** Doaa S. Farghaly, Al-Shaimaa M. Sadek

**Affiliations:** Department of Zoology and Entomology, College of Science, Al-Azhar University, P. O. 11765, Cairo 11865, Egypt

**Keywords:** diminazene aceturate, hematology, methanol extract, *Sarcophaga argyrostoma* larva, trypanocidal activity, *Trypanosoma evans*i

## Abstract

**Background and Aim:**

Many natural products worldwide are used for medicinal purposes. Various insect-isolated compounds were investigated in pursuit of new therapeutic agents. This study aimed to compare the effects of methanol extract of hemolymph of *Sarcophaga argyrostoma* larvae with diminazene aceturate on some hematological and biochemical indices of mice infected with *Trypanosoma evansi*.

**Materials and Methods:**

Sixteen albino mice were randomly divided into four groups, of four mice, which received different treatments: In Group 1 (G1), mice were infected intraperitoneally with 1×10^4^ *T. evansi* and received no treatment (positive control), in Group 2 (G2), infected mice were treated with 0.5 mL/kg of diminazene aceturate, in Group 3 (G3), infected mice were treated with 0.5 mL/kg methanol extract of the hemolymph of *S. argyrostoma* larvae, and in Group 4 (G4), uninfected mice received 0.5 ml of distilled water (negative control). In G3, treatment was started 3 days before injecting the parasite, while for the other groups, a single dose of treatment was applied when the parasite appeared in the blood.

**Results:**

Mice from G3 showed low parasitemia of 29×10^4^/mm^3^ 4 days post-infection until the infection completely disappeared on the 5^th^ day, which was earlier than for other groups. The results showed that the numbers of red blood corpuscles (red blood cells [RBCs]) and white blood cells (WBCs) per unit volume were significantly different (p<0.05) between the four groups. The highest RBC (9.09×10^3^ cell/ mm^3^) and WBC (14.30×10^3^ cell/ mm^3^) counts were recorded in G3, whereas the lowest values of 6.60 and 4.60×10^3^cell/ mm^3^, respectively, were recorded for G2. In addition, there were significant differences (p<0.05) between the different groups for platelet counts per unit volume, with G3 having the most (943×10^3^ cell/ mm^3^) and G2 having the least (357×10^3^ cell/ mm^3^). There was a significant (p<0.05) difference in the indices of biochemical activities between the extract-treated infected groups and the standard drug-treated group.

**Conclusion:**

This study suggests that the methanol extract of the hemolymph of *S. argyrostoma* larva exhibits trypanocidal activity, so it may be exploited as a suitable candidate for the development of trypanocidal drugs.

## Introduction

Animal trypanosomiasis is caused by *Trypanosoma brucei*, *Trypanosoma evansi*, *Trypanosoma congolense*, and *Trypanosoma vivax* [1] and occurs over ~25 million km^2^ in Africa. It limits the growth of the livestock industry in Africa [2] and poses a major obstacle to economic development. Therefore, it is a priority for biomedical and public agencies, the agricultural sector, and the scientific community [3].

Among mammalian trypanosomes, *T. evansi* is the most ubiquitous parasite [4]. The control of trypanosomosis over the years has broadly included two strategies, including the use of chemotherapeutic agents and vector control [5]. Chemotherapy for *T. evansi* in domestic livestock depends on restricted compounds, of which several are closely related chemically. Of these compounds, the most excessively used therapeutic agent is diminazene aceturate. At present, diminazene aceturate (DA), isometamidium, and homidium chloride are used to treat animal trypanosomosis [5]. Although the usage of diminazene is common in most African countries, many research studies have proven that there are many problems related to its use, such as DNA damage, organ damage from the hepatotoxic and nephrotoxic effects of DA residues [6], and indirect effects on the host immune system [7].

Attempts to find new trypanocidal drugs have become more or less static over the past three decades because of a lack of interest by the pharmaceutical industry to invest in research and development of new anti-trypanosomal drugs [8].

To date, the fight against this disease relies chiefly on old chemotherapy and chemoprophylaxis, which are expensive and toxic, with severe and even fatal side effects when the blood-brain barrier is crossed [9]. In addition, the appearance of drug-resistant trypanosomes aggravates the problem, which calls for more research into the development of alternative, less toxic, and more efficient drugs [1].

In traditional medicine, insect bodies, eggs, and secretions have been used to cure diseases for more than 2000 years. Recently, modern scientific studies have revealed the therapeutic functions of insect extractions, including their antibacterial, anti-inflammatory, anti-tumor, immune regulating, and blood sugar reducing properties. Numerous compounds extracted from insects have been tested as important resources for the discovery of new drugs [10].

Insects and their products are essential ingredients in the preparation of drugs in folklore medicine [11]. The housefly, *Musca domestica*, is the most common of all domestic flies, and it accounts for about 91% of all flies in human habitation. It is one of the major insect that is globally distributed [12]. Extractives from houseflies have been cited for their antioxidant [13], antimalarial [14], antibacterial, immune activation, antiviral, and antitumor properties [15]. As necrophagous flies live in an environment filled with microorganisms, they must possess robust immune cellular and humoral components to counter infection [16]. Antibacterial peptides have been isolated from the hemolymph of flesh fly, *Sarcophaga peregrina*, larvae [17].

Shittu *et al*. [14] revealed that the methanol extract of *M. domestica* maggots contains antiplasmodial activity. This finding demonstrated the significance of insects and their products for the development of new drugs for treating malaria and other infectious diseases.

Biochemical parameters are useful markers for assessing tissue damage [18]. Deficiencies in the activities of these markers mirror the level of infection and toxicity of test compounds [19], as it has been established that trypanosomal infection alters biomarker enzymes [1]. Therefore, this study compared the trypanocidal activity of methanol extracts of the hemolymph of *Sarcophaga argyrostoma* larvae and DA and their effects on some enzymes and hematological parameters in *T. evansi* infected mice.

## Materials and Methods

### Ethical approval

Animal experiments were conducted according to the requirements of the Institutional Animal Care and Use Committee, National Research Centre Animal Care Unit in Egypt, which is compatible with the guidelines of the International Animal Ethics committee (8^th^ Edition 2011) by Institute for Laboratory Animal Research; Division of Earth and Life Studies; National Research Council (Record number: 13799, legacy ID: 8247); and the local laws and regulations.

### Study period and location

The study was conducted from April to June 2019. *Sarcophaga argyrostoma* larvae were collected and maintained for several generations at the laboratory of the Zoology Department, Faculty of Science, Al-Azhar University and then extraction of hemolymph was conducted in the regional center of fungi and its application in Cairo, Egypt. The experimental part of the study was realized at Faculty of Veterinary, Assiut University, Egypt.

### Collection of *S. argyrostoma*

#### Insect colony

*S. argyrostoma* larvae were collected and maintained for several generations at the laboratory of the Zoology Department, at the Faculty of Science, Al-Azhar University, under controlled conditions of 25±5°C, 60±10% relative humidity, and a 12:12 h light:dark photoperiod. Adults were fed on 10% sucrose solution while maggots were reared on bovine meat [20].

#### Hemolymph collection

Hemolymph was collected from each group by cutting off the anterior tip of the larvae using sterile, fine scissors, and placing it in an ice-cold Eppendorf containing a few crystals of phenylthiourea to prevent melanization [21]. The hemolymph was then put through the lyophilization process following LaTorre-Snyder [22]. Then, 20 g of the powder extract was dissolved in absolute methanol in a closed glass vessel and kept in the shade for 48 h, after which it was filtered through Whatman filter paper. The filtrate was collected in a beaker, exposed to air, and allowed to evaporate for 2 weeks at room temperature (37 °C), then redissolved in dimethyl sulfoxide to yield a final concentration of approximately 10 mg/ml. The work was conducted in the regional center of fungi and its application in Cairo, Egypt.

#### Experimental animals

Sixteen male albino mice, *Mus domesticus*, with an average weight of 30-35 g were obtained from the Veterinary College at Assiut University, Egypt. Animals were examined to check that they were free of parasitic infection to avoid the production of antibodies before the experiments, and they were kept in an animal house under standard laboratory care of 21°C and 16% moisture, with water available *ad libitum* and a diet containing 20% protein, 3% fat, and 22% fiber. The mice were acclimatized for 7 days before starting the experiment.

#### Collection of blood samples

Blood specimens were collected from camels that had been imported from Sudan and were housed in the quarantine area in the Halayeb and Shalateen area (23° 7′ 54″ N 35° 35′ 8″ E), which is located on the Northeast African coast of the Red Sea. Twenty mL of blood was drawn from the jugular vein of camels under complete aseptic conditions.

#### Examination of fresh blood for T. evansi

Fresh blood was examined before the inoculation of trypanosomes in animals. A drop of blood was examined immediately under the low power of a microscope. In some cases (when the parasite was not obvious under the low power), blood was immediately centrifuged for ~15 min and the deposit was examined for the presence of trypanosomes, which are usually identified by their characteristic movements [23].

### Drug usage

DA (Berenil, Hoechst, Germany) was dissolved, prepared as aliquots following the manufacturer’s instructions, and injected intraperitoneally at a concentration of 0.5 mL/kg of body weight following MSD animal health instructions, 2020.

#### Treatment of animals

Animals were randomly divided into four groups of four mice: The first group (G1) was positive control, which was infected intraperitoneally with 1×10^4^ *T. evansi* and received no treatment, the second group (G2) was injected intraperitoneally with *T. evansi* and treated with 0.5 mL/kg of DA, the third group (G3) was injected with *T. evansi* parasite and treated with 0.5 mL/kg methanol extract, and the fourth group was the negative control, which was uninfected and received 0.5 ml of distilled water. In G3, the treatment was started 3 days before the mice were infected, and the other treatments were given as a single dose when the parasite appeared in the blood.

#### The daily follow-up

Daily examination of the blood of laboratory-infected animals was conducted using wet smears and thin and thick films to test for the presence of trypanosomes to determine parasitemia. Blood was obtained from the tail and examined under a microscope at 100× following the Pizzi-Brener method [24].

#### Blood collection and preparation

Mice were anesthetized using cotton wool soaked in chloroform, and their abdominal cavities were opened until the sternum using medical scissors. Blood samples were directly drawn from the heart using a 5 ml sterile syringe into clean, dry centrifuge tubes, which were allowed to stand for 10 min at room temperature (37°C), and then centrifuged at 3000×g for 15 min using a laboratory centrifuge (SM 800B, Surgifriend Medicals, England). Sera were carefully removed and stored frozen at −80°C until their use for biochemical analyzes. The blood used for hematological analysis was collected into heparinized sample tubes containing ethylenediaminetetraacetic acid to prevent the blood from clotting and taken for analyzes within 24 h of collection.

#### Hematological studies

Hematological parameters, including red blood cells (RBCs), platelet counts, hemoglobin (HB) levels, packed cell volume (PCV), and white blood cells (WBCs), were determined using an automated hematologic analyzer SYSMEX, KX-21 (Japan), as previously described [25].

### Biochemical analysis

Serum samples were analyzed for total protein (TP) using the biuret method, albumin (Al) using bromocresol green, glucose, and bilirubin. In addition, the activity of liver enzymes, alanine aminotransferase (ALAT) or glutamic pyruvic transaminase, aspartate aminotransferase (ASAT) or glutamic oxaloacetic transaminase, acid phosphatase (AcP), and alkaline phosphatase (ALP) was determined. All biochemical parameters were determined using a Technicon RA-2000 random access, automated analyzer. All hematological and biochemical parameters were estimated using commercial kits (Biodiagnostic Co., Dokki, Giza, Egypt), as described in the manufacturer’s instructions.

### Statistical analysis

The results were expressed as the mean±standard deviation of the mean, and the significant difference between means was evaluated using a one-way analysis of variance followed by a *post hoc* test for the comparison of significance using the Statistical Package program SPSS version 23.0 (IBM SPSS Statistics, USA). Values of p<0.05 were considered statistically significant.

## Results

### Estimation of parasitemia

Four days post-infection, all animals in the infected groups tested positive for *T*. *evansi*. The average prepatent period ranged from 2 to 3 days. Mice from G3 showed low parasitemia 29×10^4^/mm^3^ 4 days post-infection until the infection completely disappeared on the 5^th^ day, which was earlier than the other groups. The course of the parasitemia in the groups is shown in Table-1.

**Table-1:** Course of parasitemia in mice experimentally infected with *T. evansi* over the 10 days of the experiment.

Days post infection	G1 infected with *T*. *evansi*	G2 infected and treated with Berenil	G3 infected and treated with methanol extract
4	32×10^4^/mm^3^	30×10^4^/mm^3^	29×10^4^/mm^3^
5	58×10^4^/mm^3^	28×10^4^/mm^3^	Nil
6	122×10^4^/mm^3^	20×10^4^/mm^3^	Nil
7	157×10^4^/mm^3^	22×10^4^/mm^3^	Nil
8	260×10^4^/mm^3^	Nil	Nil
9	300×10^4^/mm^3^	Nil	Nil
10	320×10^4^/mm^3^	Nil	Nil

T. evansi=Trypanosoma evansi

### Hematological studies

Counts of RBCs and WBCs were significantly different (p<0.05) between the four groups (Table-2). The highest counts per unit volume of RBCs (9.09×10^3^ cell/mm^3^) and WBCs (14.30×10^3^ cell/mm^3^) were recorded in G3 followed by G4 (8.12 and 13.80×10^3^ cell/mm^3^, respectively), whereas G2 showed the lowest values for RBCs (6.60×10^3^ cell/mm^3^) and WBCs (4.60×10^3^ cell/mm^3^). For platelet counts, there were significant differences (p<0.05) between the four groups. Mice in G3 had the highest (943×10^3^ cell/mm^3^) platelet counts followed by mice in G4 (783×10^3^ cell/mm^3^) then mice in G1 (561), whereas the lowest value was recorded for mice in G2 (357×10^3^ cell/mm^3^). Similarly, the treatment had a significant (p<0.05) effect on HB and PCV concentrations: Mice in G3 showed the highest (15.40 g/dL and 37.63%) values followed by G4 (14.80 g/dL and 35.20%) then G1 (12.50 g/dL and 31.00), while the lowest value was recorded by G2 (12.30 g/dL and 29.24%) for HB and PCV, respectively (Table-2).

**Table-2:** Hematological studies of infected, treated, and control groups.

Parameter	Groups				±SEM

	G1	G2	G3	G4	
RBCs (×10^3^cell/mm^3^)	7.00^c^	6.60^d^	9.09^a^	8.12^b^	0.04
WBCs (×10^3^cell/mm^3^)	7.80^b^	4.60^c^	14.30^a^	13.80^a^	0.26
Platelets (×10^3^cell/mm^3^)	561^c^	357^d^	943^a^	783^b^	0.58
HB (g/dL)	12.50^c^	12.30^d^	15.40^a^	14.80^a^	0.06
PCV (%)	31.00^c^	29.24^d^	37.63^a^	35.20^b^	0.29

RBCs=Red blood corpuscles, WBCs=White blood cells, HB=Hemoglobin, PCV=Packed cell volume. Different letters a, b, c within the same row indicate significant differences p<0.05

### Biochemical studies

Statistical analysis revealed significant differences in TP concentration due to treatments (p≤ 0.05; Table-3): The highest (8.10 mg/dL) TP values were for mice in G3 followed by mice in G1 (7.70 mg/dL) and G4 (7.60 mg/dL), while the lowest value was recorded for mice in G2 (7.00 mg/dL; Table-3). However, mice in G4 had a higher (p<0.05) value of Al than other groups, while G3 had the highest (p<0.05) globulin concentrations (Table-3), whereas glucose and ALP concentrations were highest (p<0.05) in G1, glucose was lowest in G4 and ALP was lowest in G2 (Table-3). However, mice in G2 group had the highest concentration of AcP, which was significantly higher than that for G3 and G4 mice (Table-3).

**Table-3:** Biochemical studies of infected, treated, and control groups.

Parameter	Groups				±SEM

	G1	G2	G3	G4	
Total protein (mg/dl)	7.70^ab^	7.00^b^	8.10^a^	7.60^ab^	0.26
Albumin (mg/dL)	3.40^b^	3.20^c^	3.50^b^	4.10^a^	0.06
Globulin (mg/dL)	4.30^abc^	3.80^ab^	4.60^a^	3.50^b^	0.02
Glucose (mg/dL)	140^a^	134^b^	122^c^	120^d^	0.58
ALP (U/L)	105^a^	14.00^d^	16.00^c^	18.03^b^	0.52
ACP (U/L)	0.90^ab^	1.02^a^	0.80^b^	0.03^c^	0.04
ALAT (GPT) (U/L)	46.80^a^	45.20^a^	35.00^b^	18.00^c^	0.52
ASAT (GOT) (U/L)	146^b^	166^a^	80.00^c^	72.30^d^	0.52
Bilirubin (mg/dL)	0.62^a^	0.56^b^	0.62^a^	0.24^c^	0.01

ALP=Alkaline phosphatase, ACP=Acid phosphatase, ALAT (GPT)=Alanine aminotransferase (glutamic pyruvic transaminase), ASAT (GOT)=Aspartate aminotransferase (glutamic oxaloacetic transaminase). Different letters a, b, c within the same row indicate significant differences p<0.05

Values of ALAT (IU/L) and ASAT (IU/L) in the sera of mice were significantly different between treatments: The mice in G1 and G2 had higher (p<0.05) concentrations of ALAT and ASAT than those in G3 and G4 (Table-3). Finally, mice in G1 and G3 had higher (p<0.05) concentrations of bilirubin than those in G2 and G4 (Table-3).

## Discussion

Insects and their products constitute essential ingredients for the preparation of drugs in folklore medicine [11]. Assessments of biochemical parameters are a useful marker for assessing tissue damage [18]. Deficiencies in the activities of these markers indicate levels of infection and toxicity of test compounds [19], and trypanosomal infection alters biomarker enzymes [1].

This study demonstrates that 10 mg/ml concentrations of methanol extract of hemolymph of *S. argyrostoma* larva significantly inhibited *T. evansi* organisms *in vivo*. Similar results were recorded by Shittu *et al*. [26] using methanol extract of *M. domestica* larva. The observed inhibition may be an indication of the possible utility of the methanol extract of hemolymph of larva as an anti-trypanosomal agent.

Animal trypanosomiasis is characterized by hematological changes, which radically alter the pathogenesis of the disease in infected animals [1,27,28]. The lower concentrations of RBCs, HB, and PCV in the untreated, infected group are indications of anemia as recorded by Padmaja [29] and Eyob and Matios [30]. Decreased values of the same parameters in untreated, infected mice and infected mice treated with Berenil might be due to increased susceptibility of RBC membranes to oxidative damage [31], or as suggested by Kramer [32], anemia could be due to the depletion of erythrocytes, which might be a result of chronic liver inflammation. The increase in RBC, HB, and PCV concentrations in the infected mice treated with methanol extract suggests that the extract reduces the anemic effect of *T. evansi* in mice and helps improve hematological parameters by decreasing oxidative stress markers, as proposed by Raish *et al*. [33]. This study also showed that infection with *T. evansi* induced leukopenia and this was enhanced by treatment with methanol extract. Leukocytosis, which was recorded in our study, is a good indicator of enhancement and strengthening of the immune system by increasing the mean production of WBCs [34] and increasing mononuclear phagocytic system activity [35].

The efficiency of some organs was investigated using biochemical markers. We found that the mean values of serum TP, Al, and globulin were significantly lower in the positive control. In addition, values for these parameters were lower for the infected mice treated with Berenil than for those treated with methanol extract. The decrease in total serum protein level observed in our study is in agreement with the results of Abo-Aziza *et al*. [28], who reported a decrease in TP in camels infected with *T. evansi* and with Sivajothi *et al*. [27] who reported similar results for infected rabbits. Protein levels usually drop in trypanosome infections as a result of hepatic degeneration, damage accompanying hypoxia [36], and parasite antigens [37]. The elevations in proteins levels after treatment with methanol extract are usually due to increases in globulin levels. This is as a result of improving the immune response of the animal to *T. evansi* infection [38].

Our biochemical results revealed a marked increase in glucose in the experimentally infected animals (G1 and G2) that may be due to host organ damage at a cellular level due to toxic releases from parasites and immune complexes [36,39]. This is in accordance with Garba and Mayaki [40], who recorded high levels of glucose in donkeys infected with *T. evansi*. There was an improvement in blood glucose level in the infected mice that were treated with methanol extract (G3). This finding could be due to decreases in parasitemia levels, which lead to decreasing glucose demand by parasites [41].

As far as the activity of enzymes is concerned, ALP, AcP, ALAT, and ASAT activities showed a decrease in the group treated with methanol extract compared with the positive control group. In addition, there was a marked increase in enzymatic hepatic activity, such as ALP and AcP in untreated mice (G1) when compared with those treated with the extract (G3). Our findings are in accordance with those of Yakubu *et al*. [19]. The elevation in ALP and AcP activities in the untreated group (G1) could be attributed to infection gradually affecting enzyme levels by increasing the activation of the enzyme molecule at the site and releasing membrane components, such as alkaline and AcP, into the extracellular fluids.

Several studies have also previously reported significant increases in ALAT and ASAT activities in *Trypanosoma* sp. infected animals [26-28]. These studies explained that liver infection with *Trypanosoma* led to hepatocyte destruction and enzyme release and indicates a cellular response. However, mice treated with methanol extract of *S. argyrostoma* hemolymph showed improved enzymatic hepatic activities by significantly decreasing the serum activities of the elevated ALP, AcP, ASAT, and ALAT.

Increased quantities of total bilirubin may also indicate the increased breakdown of HB due to *Trypanosoma* spp. [42]. This result is in agreement with Gow *et al*. [43] who observed that dogs naturally infected with *T. congolense* had elevated levels of bilirubin and Sivajothi *et al*. [27] who recorded elevated bilirubin levels in rabbits infected with *T. evansi*.

## Conclusion

Our results prove that the methanol extract of the hemolymph of *S. argyrostoma* larva has trypanocidal activities. Therefore, further investigations should be conducted on insect products as a natural treatment that could be exploited as a new generation of trypanocidal agents.

## Authors’ Contributions

DSF collected the larvae of insects. AMS carried out biochemical and hematological tests. Both authors followed up the experiment, wrote and revised the manuscript, and approved the final manuscript.

## Competing Interests

The authors declare that they have no competing interests.

## Publisher’s Note

Veterinary World remains neutral with regard to jurisdictional claims in published institutional affiliation.
